# A Bayesian finite-element trained machine learning approach for predicting post-burn contraction

**DOI:** 10.1007/s00521-021-06772-3

**Published:** 2022-01-30

**Authors:** Ginger Egberts, Marianne Schaaphok, Fred Vermolen, Paul van Zuijlen

**Affiliations:** 1 Delft Institute of Applied Mathematics, Delft University of Technology, Delft, The Netherlands; 2 Research Group Computational Mathematics(CMAT),Department of Mathematics and Statistics, University of Hasselt, Hasselt, Belgium; 3 Burn Centre and Department of Plastic,Reconstructive & Hand Surgery, Red Cross Hospital, Beverwijk, Netherlands; 4 Department of Plastic, Reconstructive & Hand Surgery, Amsterdam UMC, location VUmc, Amsterdam Movement Sciences, Amsterdam, The Netherlands; 5 Pediatric Surgical Centre, Emma Children’s Hospital, Amsterdam UMC, location AMC and VUmc, Amsterdam, Netherlands

**Keywords:** Machine learning, Post-burn scar contraction, Morphoelasticity, Feed-forward neural network, Medical application, Monte Carlo simulations, 35G20, 35L65, 35M10, 35Q74, 35Q80, 35Q92, 35R37, 68T07, 74-10, 74L15, 92-10, 92B20, 92C10, 92C17, 92C45

## Abstract

Burn injuries can decrease the quality of life of a patient tremendously, because of esthetic reasons and because of contractions that result from them. In severe case, skin contraction takes place at such a large extent that joint mobility of a patient is significantly inhibited. In these cases, one refers to a contracture. In order to predict the evolution of post-wounding skin, several mathematical model frameworks have been set up. These frameworks are based on complicated systems of partial differential equations that need finite element-like discretizations for the approximation of the solution. Since these computational frameworks can be expensive in terms of computation time and resources, we study the applicability of neural networks to reproduce the finite element results. Our neural network is able to simulate the evolution of skin in terms of contraction for over one year. The simulations are based on 25 input parameters that are characteristic for the patient and the injury. One of such input parameters is the stiffness of the skin. The neural network results have yielded an average goodness of fit ($R^2$) of 0.9928 (± 0.0013). Further, a tremendous speed-up of 19354X was obtained with the neural network. We illustrate the applicability by an online medical App that takes into account the age of the patient and the length of the burn.

**Supplementary Information:**

The online version contains supplementary material available at 10.1007/s00521-021-06772-3.

## Introduction

Burn injuries are a worldwide problem. Yearly, estimates are around 180 000 deaths, and 11 million burn injuries need medical care [1]. In the long-term, burn injuries can cause reduced mobility in the burned body part because of contraction. During contraction, myofibroblasts pull on the boundary of the wound, reducing and deforming the damaged skin. Without medical care, contraction can cause lifelong disabilities affecting the patient’s quality of life. In such a case, one refers to the scar as a contracture, for which we wish to prevent its development. Burn wound dimensions (size, depth, location) and patient-specific factors (age, gender, etc.) are factors that influence contraction. This dependency is a reason for the growing interest in personalized health care.

Mathematical modeling contributes to this growing interest. Detailed models can give insight into which elements have a major influence on the contraction [2] can tune these elements and can access the uncertainty by performing Monte Carlo simulations. This allows for patient-based predictions and can help medical staff in making the optimal treatment choices. However, to achieve personalized health care, we need many model-based predictions, with the downside that high-dimensional mathematical models are expensive.

As a result, we need to use and develop alternatives to predict post-burn contractions, as it makes little sense for medical staff to wait days or weeks. Neural networks can reproduce complex relations within a short evaluation time after enough training [3]. The medical society has benefited for years from neural networks and deep learning. For example, computer vision has been used to classify skin burns [4] and to classify tumors [5]. Furthermore, neural networks have been used to find diseases, such as the coronavirus disease, in blood samples [6].

Skin is the largest organ of our body, and it is also a complicated organ. Skin typically consists of several layers: The top layer is the epidermis, the second layer is the dermis and the third layer is the subcutis. Our modeling framework has been designed for deep tissue injury in which at least the dermal layer has been damaged. We focus on post-burn skin contraction, for which we have a mathematical model. Skin contraction takes place in the dermal layer of the skin (the dermis). The displacement of the dermis generates strains, which we assume to be infinitesimally small. In short, the model comprises a system of six coupled, nonlinear partial differential equations. Four equations represent dermal constituents, and the other two represent the displacement velocity and the effective strain. The constituents’ interaction leads to a reduction in the wound size, which we describe as the relative surface area (RSA) of the damaged tissue.

In this study, we train a feed-forward neural network to predict the nonlinear mapping from the patient- and wound-specific data to the RSA. This is a common approach. For example, Yang et al. used a convolutional neural network to speed up the approximation of the stress–strain curve for materials [7]. Wang et al. considered a long short-term memory neural network to speed up mechanical models used for studying the dynamics of biological systems [8]. Navratil et al. have shown that a neural network can outperform other, non-intelligent, acceleration techniques on both acceleration and accuracy [9]. In particular, they compare neural networks to simple procedures, including up-scaling, to speed up the physics-based simulations in oil reservoir modeling. The results show a possible speedup of 2000X and two orders of magnitude reduction in average sequence error concerning the simulator.

Our goal is to speed up the predictions of post-burn scar contractions for a medical purpose. In contrast to one-dimensional models, more-dimensional models suffer from long computation times. Hence, as a preliminary study for more-dimensional models, we consider the application of a neural network for the one-dimensional model first. We create many data samples using the numerical approach by varying parameter values. Then, we fit a two-layer feed-forward neural network and assess generalization using cross-validation. To illustrate how we can make use of such a neural network in the future, we implement the optimized network in an online application.

We organized this paper as follows. Section 2 presents the mathematical model and numerical implementation, and Sect. 3 presents the neural network. Subsequently, Sect. 4 presents the results and the illustrative (medical) application. Finally, Sect. 5 presents the conclusions, and Sect. 6 presents the discussion and further work.

## The mathematical model

Our study uses the one-dimensional counterpart of the morphoelastic model for scar contraction [10]. This model simulates contraction during wound healing and scar maturation by considering a chemical response that induces the (permanent) displacement of the skin and the effective (remaining) strain. The model captures the chemical response using four species: signaling molecules (*c*), fibroblasts (*N*), myofibroblasts (*M*), and collagen ($\rho$). These equations have the general form

1 $$\begin{aligned} \dot{z} + (zv)' = - J_z' + R_z, \quad z\in \{c,N,M,\rho \}. \end{aligned}$$

Here, $\dot{z}$ denotes the time derivative of *z*, $(zv)'$ models passive convection (as the points in the domain are subject to displacement), and $J_z, R_z$ denote the flux and the chemical response of *z*, respectively. Further, the model includes the dermal displacement (*u*), the displacement velocity (*v*), and the infinitesimal effective strain ($\varepsilon$). The equation for the displacement velocity is

2 $$\begin{aligned} \rho _t \left( \dot{v} + 2vv' \right) = \sigma ' + f, \end{aligned}$$

where $\sigma$ represents the stress associated with the dermis, and *f* represents the body force working on the dermis caused by cell traction. Finally, the equation for the effective strain is

3 $$\begin{aligned} \dot{\varepsilon } + v\varepsilon ' + (\varepsilon -1) v' = -G, \end{aligned}$$

where *G* is a growth contribution that we further see in growth of tissues (such as tumors). We solve the system of differential equations using the finite element method with linear basis functions. For the time integration, we apply the backward Euler method, using a monolithic approach with inner Picard iterations to account for nonlinearity. For a complete overview of the model (including parameters), initial and boundary conditions, and further derivations of the numerical methods, we refer to our earlier study [11], as they are not essential for this study.

**Fig. 1:**
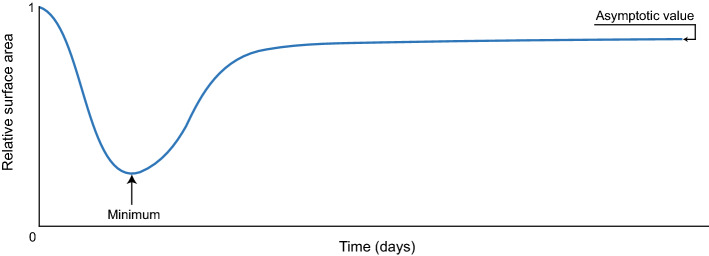
A typical relative surface area (RSA) distribution with minimum and ‘asymptotic’ values highlighted. The minimum RSA value corresponds to maximum contraction during healing, and the asymptotic RSA value corresponds to the fixed percentage of contraction after scar remodeling

***Relative surface area*** Because myofibroblasts pull on the surrounding collagen fibers, the scar contracts toward its center and retracts after these cells disappear. Figure 1 shows an example of the RSA and highlights the minimum and the asymptotic values. The minimum RSA value corresponds with the maximum contraction during healing. Once the minimum RSA has been reached, the scar retracts (i.e., myofibroblasts disappear and the scar relaxes). After remodeling, the scar does not change anymore and ends with a fixed percentage of contraction. This is the asymptotic RSA value, which we refer to as the ‘last RSA value.’

## A neural network for post-burn scar contraction

The morphoelastic model for scar contraction consists of many parameters that differ between patients and wounds. Because the model is highly nonlinear, the numerical evaluation of uncertainty in patient- and wound-specific scar contraction data is expensive. We therefore consider a feed-forward neural network to replace the numerical computations. In this section, we define the neural network applied in our study.

### Formulation

**Fig. 2:**
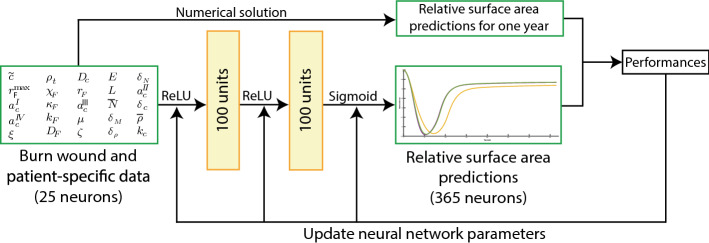
Graphical overview of our proposed feed-forward neural network

We consider a burn of length *L* cm. Together with 24 other independent parameter values, the length makes up the input vector $\mathbf{x}$. Given this input, the wound/scar changes in size over time in the course $\mathbf{y}$. Here, $\mathbf{y}$ is the non-dimensional RSA, determined by the numerical model that uses a one-day time step and 365 days as total simulation time. The goal is to learn $f(\mathbf {x};\varvec{\theta })\approx \mathbf {y}$, with $\varvec{\theta }$ the learnable parameters of the feed-forward network. In our network, we use two hidden layers with 100 neurons each and the *rectified linear unit* [12] to describe the features. On the output layer, we use the sigmoid function, because the RSA bounds between 0 and 1. We note that this output unit gives better (significant) results for our study compared with other output activation functions. Other activation functions give such poor results ($R^2<0$) that returning the expected value is a better choice. Note that, the numbers of input and output neurons are 25 and 365. Figure 2 shows a graphical overview of the method.

### Training, validating and testing

**Fig. 3:**
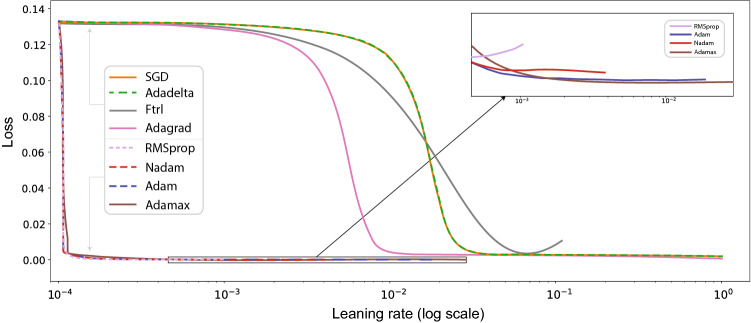
Results on the learning rate range test/loss values, showing the moving averages. The Adamax optimizer takes the largest learning rate value and provides the smallest loss. Here, the abbreviations are stochastic gradient descent (SGD), follow the regularized leader (Ftrl), adaptive gradient (Adagrad), root-mean-square propagation (RMSprop), Nesterov-accelerated adaptive moment (Nadam), and adaptive moment (Adam). Adadelta extends Adagrad, and Adamax is a variant of Adam based on the infinity norm

During the training of the neural network, we minimize the mean squared error (MSE) loss by using the Adamax algorithm with the standard backpropagation algorithm [13]. We perform a learning rate range test to discover the largest learning rate value that can train the model without divergence. We vary learning rates between 0.0001 and 1 and run for 150 epochs in batches of 64 samples. The learning rate range test takes around 12.5 minutes on a 64 bit Windows 10 Pro system with 16 GB RAM and 3.59 GHz AMD Rizen 5 3600 6-Core Processor. Figure 3 shows that the optimizers adaptive moment (Adam) and Adamax, a variant of Adam, allow larger learning rates than optimizers root-mean-square propagation (RMSprop) and Nesterov-accelerated adaptive moment (Nadam). Further, these optimizers reach better scores than optimizers stochastic gradient descent (SGD), Adadelta, follow the regularized leader (Ftrl), and adaptive gradient (Adagrad). We note that a smaller number of epochs (30) yield the same results. Given these results, we choose an initial learning rate of 0.015 with a standard decaying factor of 0.99. To avoid model overfitting, we use the early stopping regularization. We follow the MSE loss and stop training if 30 epochs show no improvement. Changes between MSE loss smaller than $10^{-5}$ are qualified as ‘no improvement.’

### Data

To train and test the neural network, we use a dataset of $n=18000$ simulations from the numerical algorithm of size $n\times 25\times 365$. This dataset is well varied, as we define a range of acceptable values for each of the input parameters that vary between patients and simulations. Based on the ranges, we define uniform statistical distributions from which we draw parameter samples. We accept samples that satisfy $k_c<\delta _c\,\overline{\rho }\,a_c^{II}$, a stability condition of the mathematical model [11]. Tables S1 and S2 in File S1 show the values for the varied parameters and the fixed parameters. Each simulation computes the results on a domain of 10 cm with a uniform spatial grid of 202 grid points. We split the large dataset into standardized (using Min-Max scaling) train- and test sets, with 80%/20% train–test split and run with tenfold cross-validation.

### Performance measures

We include the goodness of fit ($R^2$) statistic, which depends on the $L^2$ norm. Let $e_i = y_i - \hat{y}_i$ define the residual for the true (finite element) value $y_i$ and the corresponding predicted value $\hat{y}_i$. Then, $R^2=1-\sum _{i=1}^N e_i^2/\sum _{i=1}^N (y_i - \overline{y})^2$, with a positive denominator. Note a small sample standard deviation does not give lower residuals. Hence, the $R^2$ can become small (and negative) when the results of the finite element simulations show a smaller standard deviation than the mean square error. Further, we compare models $M\in \mathcal {M}$, where $\mathcal {M}$ is the set of neural networks suitable for our problem. Therefore, since $\sum _{i=1}^N (y_i - \overline{y})^2$ will stay constant among the models, maximizing $R^2$ is minimizing the square error loss, or the $L^2$ norm. Next, we include the average relative root-mean-squared error (aRRMSE), often used for (multi-target) regression problems [14]. Finally, we include the average relative error (aRelErr). Although the aRelErr is easy to interpret, this performance measure is not suitable for the entire set of targets.

## Results

**Fig. 4:**
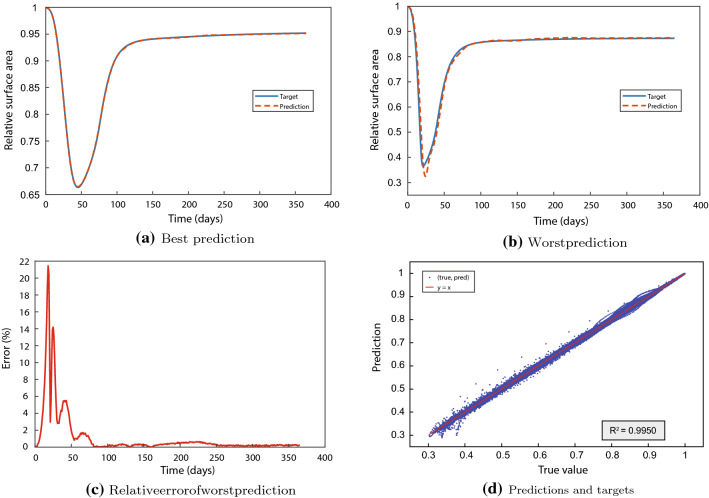
Results from the neural network for the relative surface area (RSA) prediction. The upper two graphs show the best (**a**) and worst (**b**) predictions. The lower two graphs show the relative error of the worst prediction (**c**), and the relation between the predictions and the targets, the line $y = x$ and the $R^2$ (**d**). Here, we have included the values of the entire set of time values, hence $3600\times 365$ data points

We train the neural network for predicting the RSA. Figure 4 shows the best and the worst prediction in terms of the MSE, the relative error at each point for the worst prediction, and the relation between the predicted and target values for the samples in the test set. Figure 4a shows that, in the best-case scenario, the prediction is much indistinguishable from the target. Figure 4b shows that, in the worst-case scenario, the network estimates the greatest contraction to be around 5% more intensive than the target value. The relative error of the worst prediction increases to 22% and converges to less than 1% for the final contraction intensity in Fig. 4c. Finally, Fig. 4d shows the predictions are correct, as the (target, prediction) distribution is more or less the $y = x$ line, the latter shown in red for comparison. There are outliers, both above and below the $y = x$ line. There is a dense distribution of outliers in the range $0.31\le x \le 0.39$, showing the model could be less correct for such contraction values exceeding 60%. This is consistent with Fig. 4b, c. It could be more difficult to predict these less often occurring cases.

To substantiate our observations, Table 1 shows the performance measures and the training and validation times. The cross-validation trials return a mean $R^2 = 0.9928$, with a standard deviation of 0.0013. For the test set, we obtained $R^2 = 0.9950$, which fits within the 95% interval of confidence. The $R^2$ results show accurate predictions. The aRRMSEs are 0.0626 (± 0.0080) and 0.0509 for the folds and test set. These results are smaller than 0.1 and so, according to Despotovic et al. [15], this trained neural network shows excellent reproduction of the finite element data. The aRelErrs of the predictions are only 0.23% (± 0.03%) and 0.19%, supporting our claim that the neural network can predict the RSA.

**Table 1:** Performance of the neural network for predicting contraction

Performance measure	Cross-validation value	Test value
$R^2$	0.9928 ± 0.0013	0.9950
aRRMSE	0.0626 ± 0.0080	0.0509
aRelErr	0.0023 ± 0.0003	0.0019
Training time	156 s	–
Validation time	–	0.93 s

During healing, the RSA reaches a minimum which, together with the last value, is interesting from a clinical point of view. Compared to the overall performance, focusing on these characteristics makes interpreting the values easier. Table 2 shows the $R^2$ and the mean absolute error (MAE) for both the minimum and last RSA values over the test set. We further show the general characteristics of the distributions to place the MAE in context.

**Table 2:** Performances for the minimum and the last relative surface area (RSA) values

Characteristic	$R^2$	MAE	Min	Max	Range	Average
Minimum RSA	0.9981	0.0028	0.3028	0.8095	0.5067	0.5599
Last RSA	0.9984	0.0008	0.7921	0.9649	0.1728	0.9044

The table shows the performance measures of the goodness of fit ($R^2$), the mean average error (MAE), and the minimum, maximum, range and average of the mentioned RSA values

The later predictions are better than the early predictions (not shown here). Therefore, not surprising that the $R^2$ of both the minimum and last RSA values have a larger score (0.9984 and 0.9980) than the overall performance score (0.9928). Both scores differ at least four standard deviations from the overall performance, hence Chebyshev’s Theorem states the exceeding probability to be bounded from above by 0.0625. The minimum RSA MAE is 0.55% of the range of values and 0.50% of the average value, supporting the network’s performance. However, we note that the neural network is less accurate for small values, where differences of 7.5% can occur. Overall, the network can distinguish between the minima within the range of 30 to 80%. The last RSA value MAE is 0.46% of the range of values and 0.09% of the average value. Hence, the network can predict the ‘asymptotic’ contraction intensity as well. We note the greatest last RSA value prediction absolute error is less than 0.7%. We conclude that the trained network can predict the RSA at various times and for ranges of parameter values.

Finally, the validation time is only 0.93 seconds in which the network predicts 3600 samples, hence, on average, 0.26 milliseconds per sample. This is significantly faster than the numerical model, which, on average, takes about 5 seconds per simulation (about 5 hours for our test set). Hence, the neural network provides a speedup of 19354X. This shows a spectacular acceleration the neural network achieves.

### Application of the neural network

The primary asset of the neural network is its quick prediction, a feature that medical staff needs to act on a burn right away. We assess the parameter uncertainties with Monte Carlo simulations to give insight into its effect, and to offer probabilities of contractures. Quick knowledge of such courses of contractions helps to choose the best treatment. For this, we designed a computational application to show the current network’s potential. In short, the application reads the patient- and wound-specific information, with which it decides the parameter distributions. Based on our earlier study [2], we use interpolation in literature data to find age-related parameter values. The results from the Monte Carlo simulations are post-processed and visualized in the application. We published the application on Heroku [16], a cloud application platform. The application is available at http://contraction-nn-r1.herokuapp.com/.

## Conclusions

The numerical approximations of post-burn contraction are expensive from a computational point of view, and hence less suited for applications that need many simulations. Hence, we aim at a cheap alternative modeling strategy based on a neural network. Our neural network is easy to train, and it provides quick predictions for a one-dimensional post-burn scar. On the test set, the network gives aRellErr = 0.19% and $R^2 = 0.995$. In addition, the network gives accurate predictions of the important minimum and last RSA values. For the minimum RSA, it reports MAE = 0.0028 and $R^2$ = 0.9981, and for the last RSA, it reports MAE = 0.0008 and $R^2$ = 0.9984. Further, the neural network framework is 19354 times faster than the finite element implementation. Taken together, our two-layer neural network performance is excellent. We developed a neural network-based application that takes patient- and wound-specific information. The fast computations allow for Monte Carlo-based predictions to access parameter uncertainty. The application serves as an example of how to offer clinicians immediate access to scar contraction simulations. In conclusion, the neural network is effective and cheap. In addition, it increases the application of parameter studies and patient-based health care. The goal is to optimize the treatment of post-burn contractions. If we do, clinicians can adjust therapies depending on complications that an efficient and reliable computational framework can predict.

## Discussion and further work

Parameter values depend on patient- and wound-specific characteristics, in particular, the patient’s age. We used linear interpolation to find age-related parameter values, which might be too simplistic. Inter-parameter dependency and patient-specific factors need research. For example, the skin’s elasticity differs between locations on the body [17]. Hence, the wound’s location is important. Further, the morphoelastic model needs to consider children’s growth and elderly excess skin.

Higher-dimensional models account for the wound shape and depth. The downside, however, is that such models lead to numerical computational complexity, and stability is harder to prove for the finite element method. Rotational symmetry and isogeometric analysis offer solutions to the curse of dimensionality and the dropping quality of a moving mesh. For the neural network, we can fit the wound using a convolutional neural network that takes in images of the initial wound, such as laser Doppler images. Pixel-based metrics can extract contours and features from these images. Another approach is to use shape similarity [18] and shape matching [19]. This way, we can use standard geometrical objects, such as circles and squares for which contraction prediction is less complicated. The edge error can measure such mapping’s error [20]. For these standard geometrical objects, we can make use of factors, such as shape indices.

From a computational point of view, it is interesting to study machine learning approaches that work with variable data (e.g., long short-term memory for time series forecasting). In real life, burns heal at different paces and applied treatments resolve contractures, after which a contracture can develop again. In such cases, we want to predict over a different period. Hence, hybrid approaches might be necessary to achieve this, though we need many clinical data samples to train such a model. Further, we assumed that the finite element predictions represent the true, real-life contraction. We can make these predictions as accurate as we want them to be, but they still remain approximations. Hence, to draw more detailed conclusions on the accuracy of the neural network for real-life applications, numerical accuracy needs to be studied in more detail.

## Supplementary Information

Below is the link to the electronic supplementary material.

Supplementary file1 (PDF 860 KB)
