# Supplementary material for: A Bayesian finite-element trained machine learning approach for predicting post-burn contraction
Source: Neural Comput Appl. 2022 Jan 30;34(11):8635–42. doi: 10.1007/s00521-021-06772-3 (PMC8801043; doi:10.1007/s00521-021-06772-3)
Supplement: Supplementary file 1 — Supplementary file1 (PDF 860 KB) [file 521_2021_6772_MOESM1_ESM.pdf]

Supplementary Material

This supplementary document corresponds to the article *A Bayesian finite-element trained machine learning approach for predicting post-burn contraction.* Within the paper, we describe the online medical application of our neural network. In this document, we show the medical application and the chosen parameter values.

The application requires patient- and wound-specific information shown in Supplementary Fig. S1, such as the patient's age and weight, and the wound size and location. Based our previous study, where we performed a feasibility study to investigate the influence of age on the parameter values [1], we use interpolation in literature data to find age-related parameter values. First, we define a variable called *age factor*, the patient's age divided by 100. We use this factor to find suitable mean values for the parameters and for this purpose we make use of the parameter value ranges shown in Supplementary Table S1. Although it could be possible that certain parameters behave more step-like due to, for example, puberty, we assume linear interpolation between consecutive data points provides a reasonable approximation. We only apply interpolation to age-dependent parameters and we consider if the values increase or decrease with age. Then, we perform random sampling using the normal distribution with these mean values and a fixed portion of the mean values as standard deviation. The age-independent parameters have values taken from an uniform distribution, with minima and maxima as in the chosen ranges. We restrict the wound size to be within 3 and 5 cm. We cut off values outside the ranges, which can happen because of random sampling. In total, we draw 1000 input combinations for each patient, which we scale before we feed the neural network.

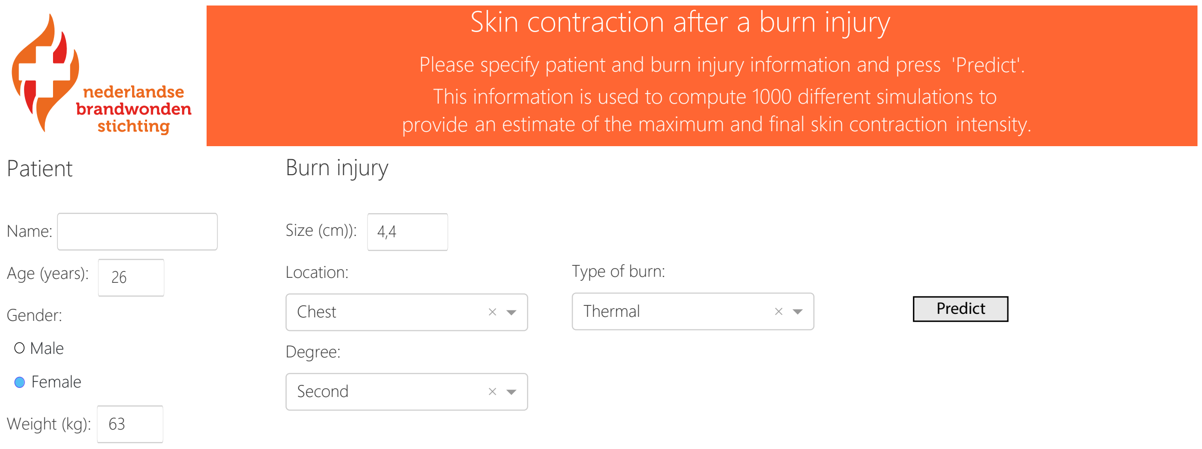

Figure S1: The input section of the application

For the predictions, we feed our trained neural network with the scaled inputs to get relative surface area predictions. Based on these predictions, we estimate the empirical cumulative probability distribution, and hence we give an estimate for the probability of developing a contracture, i.e. the probability of the final contraction exceeding a certain threshold. In the application, we show the probabilities of a maximum contraction of over 30% and the probability of a final contraction of over 10%. The user can adapt the thresholds values which recomputes the probabilities. Furthermore, the application also computes the mean relative surface area distribution, the 95%-confidence interval of the mean, and the standard deviation of the simulations from the mean. We show the mean and its confidence interval in blue, together with the interval $\mu \pm \sigma$ in red. In addition, the application also shows the histograms for the minimum and last relative surface area values. Supplementary Fig. S2 shows the visualization of the predictions for the relative surface area.

Supplementary Table S2 shows the parameter values that are kept constant.

Table S1: Ranges of varying parameter values

Parameter	Range	Dimension
$\tilde{c}$	$(1-5)\times 10^{-8}$	g/cm^3
$r_F^{\max}$	$2-3$	-
a_c^{I}	$9\times 10^{-9}-1.1\times 10^{-8}$	g/cm^3
a_c^{IV}	$8\times 10^{-10}-1.2\times 10^{9}$	g/cm^3
ξ	$(4.38-4.42)\times 10^{-2}$	(N g)/(cells cm^2)
ρ_t	$0.89-1.29$	g/cm^3
χ_F	$(2-3)\times 10^{-3}$	cm^5/(g day)
κ_F	$10^{-7}-10^{-6}$	cm^3/cells
k_F	$5.4\times 10^{6}-1.08\times 10^{7}$	cm^3/(g day)
D_F	$7\times 10^{-7}-1.2\times 10^{-6}$	cm^5/(cells day)
D_c	$(2.22-3.2)\times 10^{-3}$	cm^2/day
r_F	$0.832-0.924$	cm^{3q}/(cells^q day)
a_c^{III}	$(2-2.5)\times 10^{8}$	cm^3/g
μ	$10-1000$	(N day)/cm^2
ζ	$380-440$	cm^6/(cells g day)
E	$320-410$	N/((g cm)$^{1/2}$)
L	$3-5$	cm
$\overline{N}$	$(1-1.5)\times 10^{4}$	cells/cm^3
δ_M	$0.06-0.0885$	/day
δ_ρ	$(5.78-6.11)\times 10^{-6}$	cm^6/(cells g day)
δ_N	$0.019-0.022$	/day
a_c^{II}	$9.3750\times 10^{-9}-1.0625\times 10^{-8}$	g/cm^3
δ_c	$(4.9020-5.0980)\times 10^{-4}$	cm^6/(cells g day)
$\overline{\rho}$	$9.75\times 10^{-2}-1.25\times 10^{-1}$	g/cm^3
k_c	$(2.9605-3.0395)\times 10^{-13}$	g/(cells day)

The application is available at `http://contraction-nn-r1.herokuapp.com/`.

References

[1] Egberts G, Vermolen FJ, van Zuijlen PWM (2021) Sensitivity and feasibility of a one-dimensional morphoelastic model for post-burn contraction. Biomech Model Mechanobiol DOI 10.1007/s10237-021-01499-5. (in press)

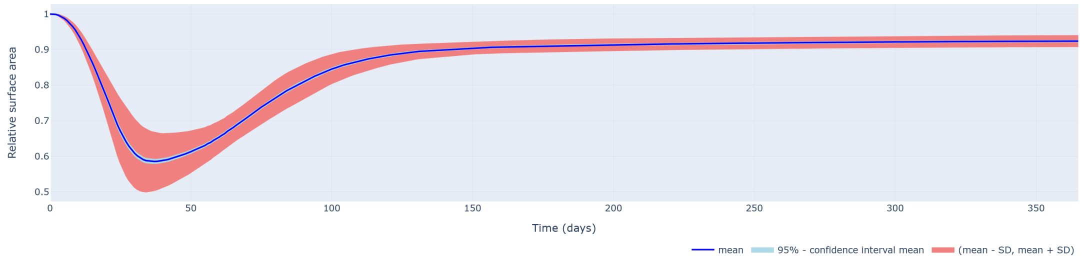

(a) Prediction of the contraction evolution in one year

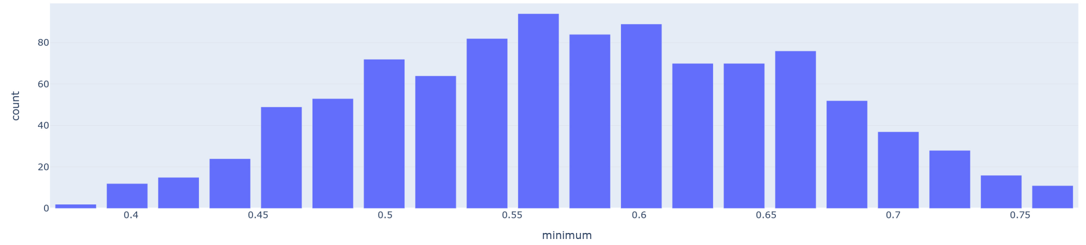

(b) Histogram of the minimum relative surface area

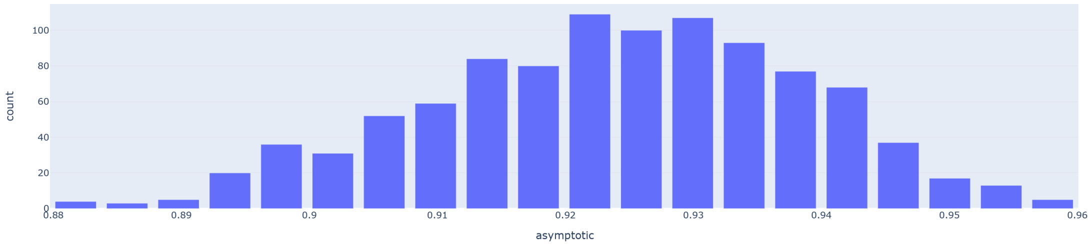

(c) Histogram of the final relative surface area

Figure S2: Visualization of the relative surface area prediction in the application. Figure S2a shows the prediction of the relative surface area in one year, Figs. S2b and S2c show the histograms of the minimum and the final relative surface area, respectively

Table S2: Fixed parameter values

Parameter	Value	Dimension
$k_\rho^{\max}$	10	-
η^I	2	-
η^{II}	0.45	-
R	0.995	g/cm^3
$\tilde{\rho}$	0	g/cm^3
